# High-resolution structural analysis of enterovirus-reactive polyclonal antibodies in complex with whole virions

**DOI:** 10.1093/pnasnexus/pgac253

**Published:** 2022-11-04

**Authors:** Aleksandar Antanasijevic, Autumn J Schulze, Vijay S Reddy, Andrew B Ward

**Affiliations:** Department of Integrative, Structural and Computational Biology, The Scripps Research Institute, La Jolla, CA 92037, USA; International AIDS Vaccine Initiative Neutralizing Antibody Center, Collaboration for AIDS Vaccine Discovery (CAVD) and Scripps Consortium for HIV/AIDS Vaccine Development (CHAVD), The Scripps Research Institute, La Jolla, CA 92037, USA; Department of Molecular Medicine, Mayo Clinic College of Medicine, Rochester, MN 55905, USA; Department of Integrative, Structural and Computational Biology, The Scripps Research Institute, La Jolla, CA 92037, USA; Department of Integrative, Structural and Computational Biology, The Scripps Research Institute, La Jolla, CA 92037, USA; International AIDS Vaccine Initiative Neutralizing Antibody Center, Collaboration for AIDS Vaccine Discovery (CAVD) and Scripps Consortium for HIV/AIDS Vaccine Development (CHAVD), The Scripps Research Institute, La Jolla, CA 92037, USA

**Keywords:** cryoEM, coxsackievirus, polyclonal antibodies, vaccine design

## Abstract

Non-polio enteroviruses (NPEVs) cause serious illnesses in young children and neonates, including aseptic meningitis, encephalitis, and inflammatory muscle disease, among others. While over 100 serotypes have been described to date, vaccine only exists for EV-A71. Efforts toward rationally designed pan-NPEV vaccines would greatly benefit from structural biology methods for rapid and comprehensive evaluation of vaccine candidates and elicited antibody responses. Toward this goal, we introduced a cryo-electron-microscopy-based approach for structural analysis of virus- or vaccine-elicited polyclonal antibodies (pAbs) in complex with whole NPEV virions. We demonstrated the feasibility using coxsackievirus A21 and reconstructed five structurally distinct pAbs bound to the virus. The pAbs targeted two immunodominant epitopes, one overlapping with the receptor binding site. These results demonstrate that our method can be applied to map broad-spectrum polyclonal immune responses against intact virions and define potentially cross-reactive epitopes.

Significance Statement Viruses with icosahedral capsids (e.g. enteroviruses, flaviviruses, adenoviruses, etc.) are a relatively large group of pathogens that infect humans and other mammals. The capsid represents the main target for antibody-mediated immune responses, and therefore the subject of vaccine design strategies. Herein, we introduce a cryo-electron-microscopy-based method for the simultaneous structural analysis of diverse polyclonal antibodies isolated from immune sera in complex with whole virions. We demonstrate feasibility using coxsackievirus A21 and mouse polyclonal antibodies. The method is generalizable and can be applied for antigenic evaluation of other icosahedral viruses and vaccine candidates.

## Introduction

Non-polio enteroviruses (NPEVs) from the family of *Picornaviridae* are nonenveloped viruses composed of (+) ssRNA genomes that infect humans and cause a variety of human diseases (1, 2). Rhinoviruses (RVs), coxsackieviruses (CVs), echoviruses, and recently emergent EV-A71 and EV-D68 are examples of viruses belonging to this family. NPEVs account for 10–15 million infections each year, particularly among neonates and young children, that result in various illnesses ranging from aseptic meningitis, viral encephalitis, acute diarrhea with echovirus 30 (E30), hand, foot, and mouth disease, and severe respiratory illness to inflammatory muscle disease that is closely associated with acute flaccid myelitis (3, 4). Over 100 different serotypes of NPEVs have been identified and these numbers are likely to increase due to the high intrinsic mutation rates during the viral replication as well as greater recombination rates between different strains. Despite a relatively large number of circulating NPEVs, no effective vaccines or therapeutics are available except for the EV-A71 vaccine approved for use in some Asian countries (5). There is a need for new therapy and vaccine solutions, particularly the ones capable of targeting multiple NPEVs instead of each one individually.

Structural biology methods, such as cryo-electron microscopy (cryoEM), have been widely used for characterization of vaccine candidates and vaccine-elicited monoclonal antibodies (mAbs) (6–8). However, these studies have been impeded by the relatively low throughput (one sample can be analyzed at a time) and the need for mAb isolation. EM-based polyclonal epitope mapping (EMPEM) is a tool for rapid and comprehensive structural analysis of immune complexes featuring vaccine-elicited polyclonal antibodies (pAbs) purified from sera (9–12). Additionally, we have recently shown that structural information from the reconstructed high-resolution maps can be used to identify sequences of polyclonal antibody families (13). EMPEM has thus far been only applied to recombinantly produced minimal antigens. In the case of enteroviruses, this is not possible, given the difficulty of producing individual capsid proteins (VP1–4), nor is it desirable, because the majority of elicited antibodies have been found to target quaternary epitopes on the viral surface (14–16).

Herein, we introduce an EMPEM pipeline for rapid structural analysis of pAb responses elicited by enteroviruses using whole viral particles. Our data processing workflow enabled reconstruction of immune complexes featuring structurally distinct pAbs at near-atomic resolution. In a case study using the coxsackievirus A21 (CV-A21), we discovered two immunodominant sites on the surface of CV-A21 readily targeted by antibodies: one in immediate proximity to the receptor binding site. Overall, we demonstrate the feasibility of this approach and provide valuable insights for future vaccine design efforts.

## Results

**Fig. 1.**
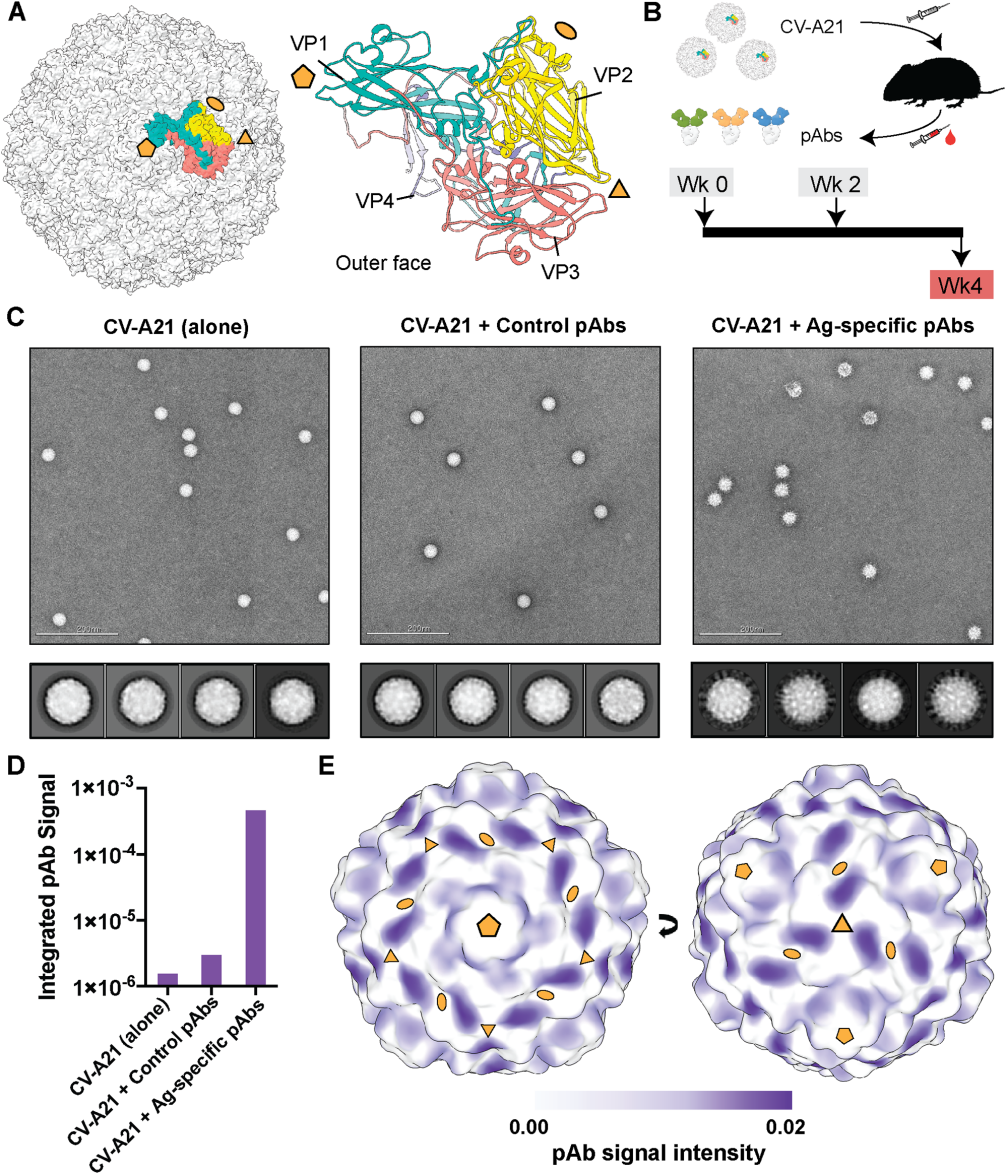
Immunization experiments and nsEMPEM analysis of polyclonal immune complexes. (A) Assembly of the CV capsid (left) and structure of the asymmetric unit (right). CV-A21 structure [PDB ID 1z7s (23)] was used to make the figure. VP1–4 are colored differently and labeled for clarity. (B) Schematic representation of the workflow used to generate pAb samples (top) and immunization regimen details (bottom). (C) Representative EM micrograph (top) and 2D class averages (bottom) of CV-A21 viral particles alone (left) or in complex with pAbs (as Fab fragments) isolated from CV-A21-naïve mice (middle) or mice immunized with CV-A21 (right). (D) Integrated pAb-corresponding signal in 3D maps reconstructed using the nsEM data presented in panel (C). These data are generated using Volume Tools in UCSF Chimera (24). (E) pAb-corresponding signal plotted on the surface of low-pass filtered map of CV-A21 viral capsid. Higher signal intensity corresponds to higher pAb occupancy. The icosahedral symmetry axes in panels (A) and (E) are indicated with golden-yellow pentamers (5-fold), triangles (3-fold), and ovals (2-fold).

For this proof-of-concept study, we selected CV-A21 viral strain responsible for a substantial proportion of enterovirus-associated acute respiratory tract infections in humans (17, 18). Additionally, this virus is evaluated clinically for its oncolytic potential (19, 20). CV-A21 and other NPEVs share the same overall icosahedral capsid structure consisting of 60 copies of VP1–4 that are arranged with pseudo, *T* *=* 3 symmetry (Fig. 1A) (21–23). Each of the VP1–3 protein subunits displays compact tertiary structure comprising a jelly-roll β-sandwich, which form the bulk of the capsid and the outer surface, making these subunits the primary targets for antibodies (Fig. 1A). VP4 adopts an extended conformation and is largely located on the inner surface of the capsid.

We sought to structurally characterize the immunogenicity of CV-A21 virus and identify primary immunogenic epitopes using a mouse animal model. Purified CV-A21 viral particles (Supplementary Material Appendix—Fig. S1A and B) were injected into mice (Fig. 1B). A control group of mice received PBS vehicle at the corresponding time points. Serum was harvested at week 4, following two antigen doses. For structural characterization, we isolated polyclonal antibodies from pooled, heat-inactivated mouse serum and cleaved them into Fab and Fc fragments with papain. Digested Fab/Fc samples were complexed with formaldehyde-treated CV-A21 particles and subjected to a round of size exclusion chromatography (SEC) to purify the viral fraction from unbound Fab/Fc (Supplementary Material Appendix—Fig. S1C). Inactivation with formaldehyde was necessary to execute experiments under biosafety level 1 conditions.

Assembled immune complexes (or free virions) were first characterized using negative stain EM (nsEMPEM; Fig. 1C). pAbs isolated from the control group of mice did not bind to CV-A21, based on the combined analysis of the SEC data, raw EM images, and 2D class averages. Conversely, antibodies isolated from animals infected with CV-A21 bound the antigen, as evidenced by the strong shift of virus-corresponding peak in SEC (Supplementary Material Appendix—Fig. S1C) and a “cloud” of pAbs (as Fab fragments) surrounding viral particles in EM images and 2D class averages (Fig. 1C, right).

We reconstructed 3D maps from particles in the above-mentioned datasets and performed partial signal integration in UCSF Chimera [Fig. 1D, Supplementary Material Appendix—Fig. S1D (24)]. pAb-corresponding signal in the polyclonal sample from CV-A21-infected mice was higher by ∼2 orders of magnitude compared to the control pAb sample, further supporting the observations made on the level of raw images and 2D. Three-dimensional maps were further used to map epitopes on CV-A21 particle targeted by pAbs (Fig. 1E, Supplementary Material Appendix—Fig. S1D). Normalized map signal above noise threshold was observed at two sites, near the 3-fold symmetry axis (Site-1) and near the 5-fold symmetry axis (Site-2). pAb cloud at Site-2 is more diffuse and less intense compared to Site-1, indicating greater diversity in antibody response.

**Figure 2.**
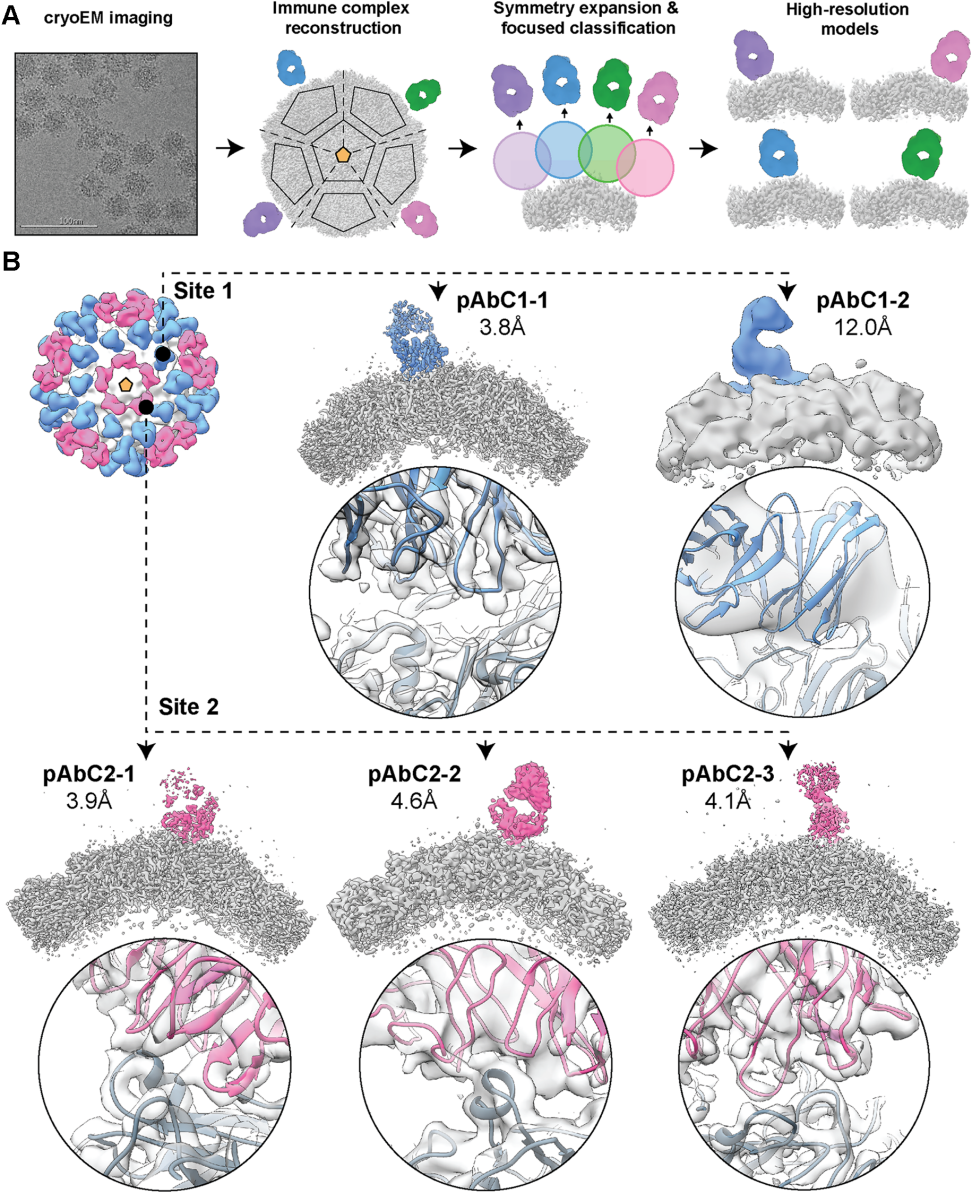
cryoEMPEM analysis of bound polyclonal antibodies. (A) Schematic illustration of the cryoEMPEM workflow. (B) High-resolution antigen-pAb immune complexes featuring structurally unique antibody specificities detected in cryoEMPEM dataset. Antigen is represented in gray and pAb densities are colored according to the epitope (blue and pink colors are used to represent Sites 1 and 2, respectively). The apparent global resolution of each reconstructed cryoEM map is indicated. Close-up views of the antibody–antigen interface are shown below each reconstructed map (cryoEM map—transparent gray surface; antigen—dark gray; antibodies—blue or pink). Ribbon representation is used for atomic models. The most relevant icosahedral symmetry axes are indicated with golden-yellow pentamers (5-fold), triangles (3-fold), and ovals (2-fold).

To further assess the diversity of polyclonal antibodies and precisely map epitope contacts, we subjected CV-A21 immune complexes to high-resolution cryoEM-based polyclonal epitope mapping [cryoEMPEM (11)] (Fig. 2A, Supplementary Material Appendix—Fig. S2, Table S1). Briefly, the vitrified immune complexes were imaged using cryoEM and particles were aligned with icosahedral symmetry restraints imposed to obtain the initial reconstruction of the entire immune complex. Prealigned particles were then symmetry-expanded where every icosahedrally related protomer is rotated and aligned onto the reference protomer, thus increasing the initial dataset by 60-fold. This allowed positioning of masks around different epitopes in a single protomer and classification of subparticles based on structural properties of bound pAbs (i.e. to combine particles with structurally similar pAbs into unique subsets). Through iterative rounds of focused classification, we reconstructed maps featuring structurally distinct polyclonal antibodies bound to epitopes at near-atomic resolution (structurally distinct polyclonal antibody class—pAbC).

We positioned spherical masks around five epitope clusters (Supplementary Material Appendix—Fig. S3A). Antibodies were only detected at Site-1 and Site-2 clusters, consistent with the nsEM data. We resolved two distinct polyclonal antibodies targeting Site-1 and three against Site-2 (Fig. 2B, Supplementary Material Appendix—Fig. S3B). The highest achieved global map resolution was 3.8 Å (pAbC1-1, Supplementary Material Appendix—Fig. S4A), although the local resolution was worse for the pAb-corresponding part of the map (Supplementary Material Appendix—Fig. S4B). Specific epitope contacts and angle of approach were highly similar for Site-1 antibodies while Site-2 pAbs displayed greater diversity (Supplementary Material Appendix—Fig. S3B); consistent with nsEM analysis.

We next relaxed atomic models into the pAbC1-1, pAbC2-1, and pAbC2-3 cryoEMPEM maps. Given the polyclonal nature of bound antibodies and the inherent lack of sequence information, the variable Fab domains (Fv) were represented as poly-Alanine models. The models were not built for pAbC1-2 and pAbC2-2 due to relatively low map resolutions. Instead, we docked mock antibody [PDB ID: 3i9g (25)] and previously published CV-A21 structure [PDB ID:1z7s (23)] into these maps (Supplementary Material Appendix—Table S2). In the case of pAbC1-2, lower global map resolution was caused by the relatively small number of starting particles (i.e. low prevalence of this antibody in the polyclonal mix), whereas for pAbC2-2, the most likely explanation is that this polyclonal complex features a higher degree of compositional and/or conformational heterogeneity. CryoEMPEM maps and corresponding models were used to assign specific epitope contacts for Site-1 (Supplementary Material Appendix—Fig. S5) and Site-2 pAbs (Supplementary Material Appendix—Fig. S6).

**Fig. 3.**
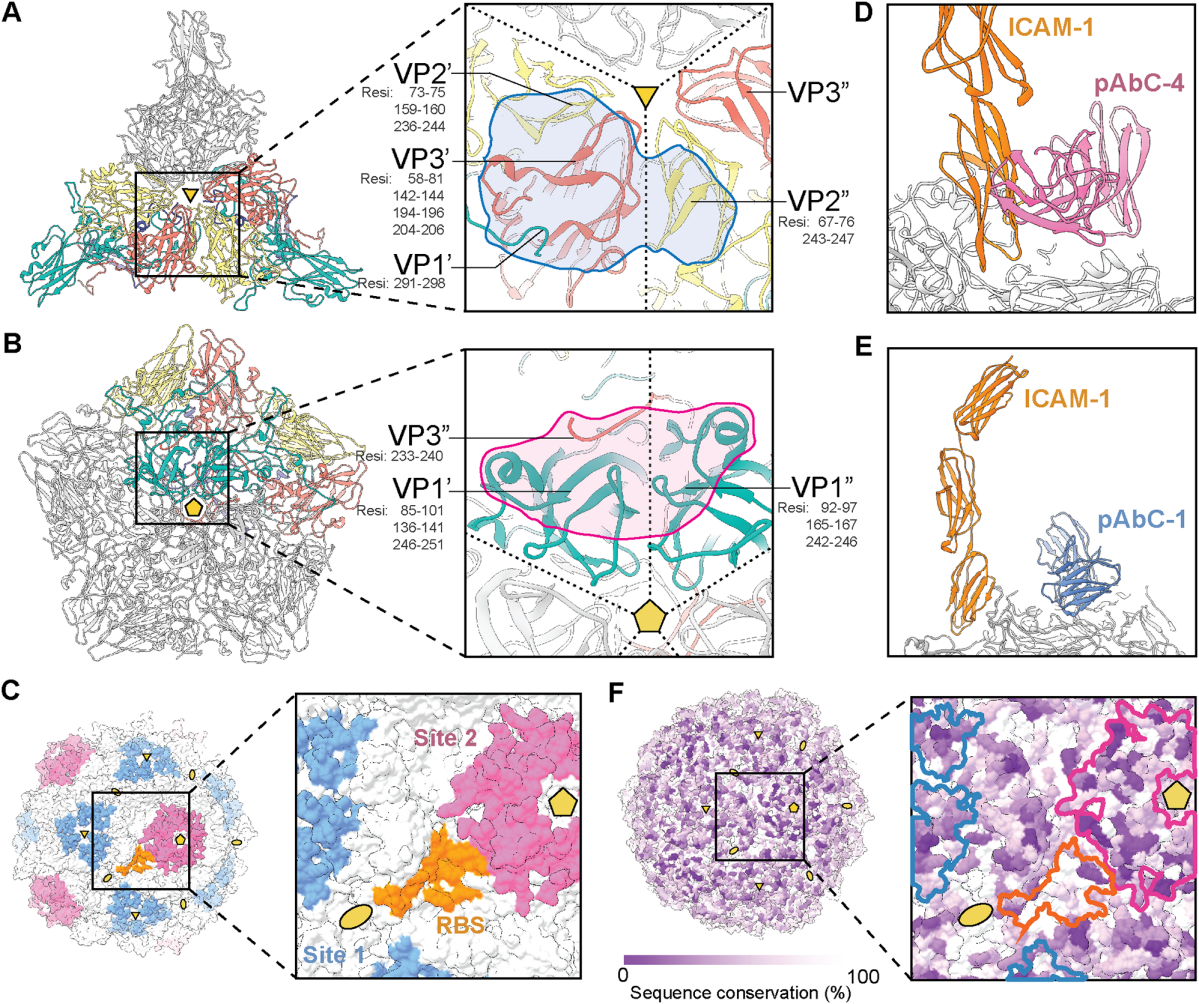
Antibody binding analysis based on cryoEMPEM data. (A and B) Locations of the Site-1 (A) and Site-2 (B) epitope clusters. Three VP protomers are shown for Site-1, and five protomers are shown for Site-2. For clarity, epitopes are presented only on a single protomer–protomer interface and the protomers comprising the interface are colored based on the specific VP subunit (VP1–green; VP2–yellow; VP3–salmon; VP4–dark blue). VP1’–VP4’ belong to the first protomer in the interface and VP1”–VP4” to the second protomer. Specific epitope residues are indicated below the chain ID. Ribbon representation is used in the panel. Polyclonal antibody footprints are indicated in blue (Site-1) and pink (Site-2). (C) Surface representation of the CV-A21 viral particle with Site-1 (blue), Site-2 (pink), and the receptor binding site (RBS; orange) indicated. For clarity, only a single RBS is highlighted. (D and E) Overlay of pAbC1-1 (D) and pAbC2-2 (E) antibody structures and ICAM-1 receptor structure [PDB ID: 1z7z; (23)]. (F) Surface representation of the CV-A21 viral particle colored based on sequence conservation. The location of the Site-1, Site-2, and RBS is outlined in blue, pink, and orange, respectively. The most relevant symmetry axes are indicated with golden-yellow pentamers (5-fold), triangles (3-fold), and ovals (2-fold).

Antibodies to Site-1 and Site-2 use different combinations of VP components for binding. Site-1 comprises VP1, VP2, and VP3 elements, while Site-2 is primarily formed by VP1 with small contribution from the C-terminus of VP3 (Fig. 3A and B, Supplementary Material Appendix—Figs. S5 and S6). pAbC1-1 and pAbC2-3 make contact with residues in both protomers and require a fully assembled viral particle to bind (Supplementary Material Appendix—Figs. S5 and S6). pAbC1-2, pAbC2-1, and pAbC2-2 interact with residues within a single protomer, but from different VP components. Importantly, all pAbs bind in a manner that does not block the accessibility to the symmetry-related neighboring binding site. In theory, all three epitopes surrounding the 3-fold symmetry axis and all five epitopes at the 5-fold symmetry axis could be simultaneously occupied with antibodies.

Site-2 is particularly interesting because it is in immediate proximity to the receptor binding site (RBS) for CV-A21 (Fig. 3C). While antibodies targeting Site-1 are distal from the RBS and it is unlikely that they would interfere with receptor binding as Fabs (Fig. 3E), antibodies against Site-2 sterically block ICAM-1 from accessing the RBS (Fig. 3D) and may be able to suppress viral entry. We then looked at the sequence conservation across all enterovirus C CV-A strains: A1, A11, A13, A17, A18, A19, A20, A21, A22, A24 (Fig. 3F, Supplementary Material Appendix—Table S3). While both Site-1 and Site-2 are highly immunogenic, the sequence conservation is relatively low, and elicited antibody responses are unlikely to cross-react with other strains of CV.

We compared the pAbs identified in this study to the structures of previously isolated mAbs targeting different CV strains (structural data is available for mAb complexes with A6, A10, A16, and B1 viruses). Overall, we observed a high degree of similarity in epitopes for several of the antibodies. For example, Site-1 overlaps with the binding sites of 14B10 (15), 5F5 (16), and 2G8 (26) mAbs; the overlay of pAbC1-1 and 14B10 mAb structures is shown in the Supplementary Material Appendix—Fig. S7A. Site-2 pAbs display high similarity in epitope and angle of approach to 1D5 mAb (14), and a partial overlap with 18A7 mAb (15) that binds directly on top of the 5-fold symmetry axis (Supplementary Material Appendix—Fig. S7B). These findings suggest that there may be a level of plasticity in mouse antibody responses to CV with respect to the locations of immunodominant epitopes. However, additional studies are necessary to establish if this is reproducible in other animal models featuring more complex B-cell repertoires.

## Discussion

Here, we introduce a method for analysis of polyclonal antibody responses elicited by nonenveloped viruses with an icosahedral capsid. We validated the approach on the coxsackievirus A21 using polyclonal antibodies from mice. In nonenveloped icosahedral virions, antibody epitopes are likely to be comprised of amino acids from multiple protomers and/or subunits. Thus, intact viral particles with native antigen presentation, instead of minimal subunit antigens, are necessary for comprehensive evaluation of elicited antibody responses. Our EMPEM methods can now be readily implemented to other nonenveloped icosahedral viruses (e.g. papillomaviruses, parvoviruses, adenoviruses), as well as enveloped viruses with icosahedral lattice on their surface (e.g. flaviviruses, togaviruses).

From a practical standpoint, the application of high-symmetry viral particles with relatively small diameter (∼30 nm) allows one to generate relatively large particle subsets after symmetry expansion (by a factor of 60). For comparison, while the diameter of homotrimeric glycoprotein spikes from flu (HA), HIV (Env), and SARS-CoV-2 (S) is ∼10 to 15 nm, the increase in particle subsets after symmetry expansion is only 3-fold. Larger starting datasets increase the number of potential pAb-epitope combinations that can be analyzed in parallel, improve the chances of finding low abundance pAbs, and increase the resolution that can be achieved.

Using a combination of nsEM- and cryoEM-based polyclonal epitope mapping, we identified two primary immunogenic sites on the surface of CV-A21 located near the 3-fold and 5-fold axes. All reconstructed antibodies bind in a manner that allows equivalent neighboring sites on the icosahedral lattice to be simultaneously occupied by the same type of antibody and achieve maximum stoichiometry of 60:1 (Fab to virus). The relative abundances of these epitopes on the CV-A21 viral particle are three or five times higher compared to epitopes lying directly on the 3-fold and 5-fold symmetry axes, respectively; providing a plausible explanation for the observed distribution of antibody responses and immunodominance of Sites 1 and 2. We speculate that this effect is further augmented by the differential capacities to crosslink B-cell receptors (BCRs). On the example of human papillomavirus (HPV), it was established that epitope spacing of ∼50 to 100 Å is optimal for BCR crosslinking and induction of robust antibody responses (27). Average distances between the nearest symmetry-related Site-1 and Site-2 epitopes are ∼30 to 60 Å while the corresponding distances for binding sites directly at the 3-fold and 5-fold axes are ∼100 and 150 Å, respectively. Consequently, there is a higher number and greater diversity of Site-1 and Site-2 epitopes within the distance range defined for HPV. Consistent with this theory, the majority of isolated monoclonal antibodies against different coxsackievirus strains (with existing structural data) target similar epitopes. The exceptions are 18A7 mAb with an epitope directly on the 5-fold symmetry axis and NA9D7 mAb interacting with the receptor binding site (15). Using EMPEM, we can study polyclonal antibody responses in a longitudinal manner enabling us to understand the hierarchy of immunodominant epitopes in different CV strains and to identify optimal vaccine candidates for eliciting the most desirable antibody responses.

Image classification in our workflow is performed locally at each epitope–paratope interface, allowing to identify/separate subpopulations of particles with dissimilar antigenic features (e.g. conformational states, bound lipid moieties). This enables the study of enteroviruses and other complex antigens that undergo structural rearrangements in response to antibody binding or other stimuli (28). Of course, the conditions for immune complex assembly would have to be optimized (e.g. incubation time, antibody–antigen ratio, etc.) to avoid material loss due to antibody-induced aggregation or disassembly of the antigen.

Antibodies targeting epitopes near the 5-fold axis can sterically block access to the receptor binding site and thereby prevent the virus from infecting cells. This feature is highly desirable, but the relatively low level of sequence conservation in this area is problematic as elicited antibodies are unlikely to cross-react with other coxsackievirus strains and may sterically compete with more desirable antibodies. Traditionally, this is achieved by immunogen engineering with many examples from the RSV, flu, HIV, and SARS-CoV-2 fields, or through viral cocktail immunizations as done with HPV and Dengue vaccines (28–36). Similar vaccine design strategies can be employed to coxsackieviruses and EM methods presented in this study open the door for rapid assessment of new vaccine candidates.

## Materials and methods

### Preparation of coxsackievirus A21 viral particles

Coxsackievirus A21 particles were propagated in H1-HeLa cells. The virions were harvested from lysed cells 2 days after infection, clarified by centrifugation, 0.2 μm filtered, and inactivated by incubation with 100 μg/mL formaldehyde at 37ºC for 3 days, followed by low-speed centrifugation to remove cell debris. The supernatant was pelleted through a 30% sucrose cushion via ultracentrifugation at 175,000 *g* for 14 hours at 4ºC. The resuspended pellet was sedimented through a 15% to 45% (w/v) sucrose density gradient and centrifuged at 120,000 *g* for 14 hours at 4ºC. Fractions containing virus particles were collected and dialyzed against PBS buffer. Inactivated particles were characterized and used for EMPEM and high-resolution structural analysis using cryoEM. The efficacy of formaldehyde treatment on virus infection was evaluated by the infectivity assay visualizing cytopathic effects in comparison to untreated controls. Total protein in virus fractions was determined and the presence of viral proteins visualized by Western blot. For structural studies, the gradient purified viral particles were polished by size-exclusion chromatography using the HiPrep 16/60 Sephacryl S-500 HR column (Sigma Aldrich) running in TBS buffer (Alfa Aesar). Fractions corresponding to coxsackievirus A21 particles were pooled and concentrated to 0.5 mg/mL using Amicon Ultra centrifugal filter units with 100 kDa cutoff (Millipore Sigma).

### Mouse immunization experiments

Animal experiments were performed at Mayo Clinic with permissions from the Mayo Clinic Institutional Animal Care and Use Committee with the registration number A00005024. C57Bl/6 mice were injected intravenously with PBS (*n* = 6) or 1 × 10^6^ TCID_50_ of purified CV-A21 virus on days 0 and 14 (*n* = 20). A serum sample was collected to verify the presence of neutralizing antibodies prior to euthanizing all mice. At the time of euthanasia (week 4), serum was collected from all immunized mice, pooled, heat-inactivated at 56ºC for 30 minutes and stored at −80ºC. Immunologically, naïve mouse serum samples were obtained and processed similarly.

### Preparation of polyclonal antibody samples

Approximately 3 mL of serum from CV-A21-immunized or naïve mice was diluted in 97 mL of PBS and ran over a column prepacked with CaptureSelect IgG-Fc (Multispecies) Affinity Matrix (ThermoFisher Scientific). IgGs were eluted with 0.1 M glycine buffer (pH 3.0) and immediately neutralized with 1 M Tris-HCl pH 8. IgG samples were concentrated and buffer-exchanged to digestion buffer (PBS + 10 mM EDTA + 10 mM cysteine, pH 7.4) using Amicon Ultra centrifugal filter units with 10 kDa cutoff (Millipore Sigma). The digestion into Fab/Fc fragments was performed for 5 hours at 37ºC using 40 µg of activated papain per 1 mg of IgG. Nondigested IgG was removed by size-exclusion chromatography, using the HiLoad 16/600 Superdex 200 pg column (GE Healthcare) running in TBS buffer. Fractions corresponding to the Fab/Fc fragments were pooled and concentrated using Amicon Ultra centrifugal filter units with 10 kDa cutoff. Final Fab/Fc yields were ∼10 mg from the starting ∼3 mL of serum.

### Preparation of immune complexes

For negative stain EM experiments, we prepared the immune complexes by combining 50 µg of purified, inactivated CV-A21 viral particles with (1) TBS, (2) 500 µg of Fab/Fc from the immunologically naïve mice, or (3) 500 µg of Fab/Fc from the mice immunized with CV-A21. The assembly reactions were incubated at room temperature for ∼18 hours, and then the samples were subjected to size-exclusion chromatography using Superose 6 Increase 10/300 GL column running in TBS buffer. Fractions corresponding to the CV-A21 virions (or immune complexes with pAbs) were pooled and concentrated using Amicon Ultra centrifugal filter units with 100 kDa cutoff.

For cryoEM, the immune complexes were assembled by combining 300 µg of the purified, inactivated CV-A21 viral particles with 8 mg of Fab/Fc from the mice that received CV-A21 virus as immunogen. Further processing was done as described in the previous paragraph.

### Negative stain EM—grid preparation, imaging, and data processing

Samples featuring CV-A21 particles (free or as part of an immune complex) were diluted in TBS to 50 μg/ml and loaded onto in-house made carbon-coated 400-mesh copper grids (glow-discharged at 15 mA for 25 second). The sample was blotted off after 10 seconds and the grids were stained with 2% (w/v) uranyl formate for 60 second. Grids were imaged on a Tecnai F20 electron microscope (FEI) equipped with a TemCam F416 CMOS camera (TVIPS). The microscope operates at 200 kV. The imaging defocus was set to 1.5 µm and the total electron dose adjusted to 25 e^−^/Å^2^. The magnification was set to 62,000×, with the resulting pixel size of 1.77 Å. Leginon software (37) was used for image acquisition and all the early processing steps were performed in Appion (38). 2D/3D classification and 3D refinement steps were done in Relion/3.0 (39). Icosahedral symmetry was imposed for the 3D classification and refinement steps. Negative stain EM maps have been deposited to the EM Data Bank under accession ID EMD-26065.

### CryoEM—grid preparation

Immune complexes comprising inactivated CV-A21 viral particles and CV-A21-elicited polyclonal antibodies were concentrated to 2.5 mg/ml using Amicon Ultra centrifugal filter units with 100 kDa cutoff (Millipore Sigma). Cryo-grids were prepared on a Vitrobot mark IV (Thermo Fisher Scientific). Vitrobot settings were as follows: temperature = 10ºC; humidity = 100%; blotting force = 0; wait time = 10 second; and blotting time varied in the 3 to 6 second range. Quantifoil R 2/1 holey carbon copper grids (EMS) were used for sample vitrification. Prior to sample application, the grids were subjected to plasma cleaning (Ar/O_2_ gas mixture; Solarus 950 plasma cleaner, Gatan). Three microliters of the sample was loaded onto plasma-cleaned grid on Vitrobot. Following the blotting step, the grids were plunged into liquid-nitrogen-cooled liquid ethane.

### CryoEM—imaging and data processing

Samples were imaged on FEI Titan Krios electron microscope (Thermo Fisher) operating at 300 kV. The microscope was equipped with the K2 summit detector (Gatan) running in counting mode. Nominal magnification was set to 130,000× with the pixel size of 1.045 Å (at the specimen plane). Automated data collection was performed using Leginon (37). Data collection information is provided in the Supplementary Material Appendix—Table S1. Raw micrograph frames were aligned and dose-weighted using MotionCor2 (40). Micrographs were then imported to Relion/3.0 for further processing (39). CTF estimation was performed using GCTF (41). Particles were picked manually and subjected to one round of 2D classification to remove bad picks. Selected particles were subjected to a round of 3D refinement with icosahedral symmetry imposed. Low-resolution map of the CV capsid was used as an initial model for the initial 3D refinement/classification steps. A tight mask around the CV capsid was applied to remove the signal contributions from pAbs and internal viral components during refinement. Prerefined particles were symmetry-expanded (icosahedral symmetry). This increased the size of the dataset by 60-fold and collapsed all bound pAbs onto every symmetry-related protomer, thus facilitating the classification of different pAbs. To avoid the possibility of symmetry-related copies from aligning to themselves, we restricted the movement of particles during the subsequent 3D refinement and classification steps. Particle alignment was suspended for 3D classification (—skip_align, T 16), and only local angular searches were allowed for 3D refinement (1.8º angular step per iteration,—healpix_order 4—auto_local_healpix_order 4). For classification, spherical masks (80 Å diameter) were positioned around different potential pAb epitopes on a single protomer (two spots) as well as directly on the 2-fold, 3-fold, and 5-fold symmetry axes. In the first round, separate 3D classifications were performed for each of the six masks used (sorted into 40 3D classes). pAb-containing classes with unique structural features (i.e. epitope and orientation) were selected and treated independently after this step. For each of the selected particle subsets, we extracted subparticle regions centering on the pAb. This was done to reduce the alignment bias from the CV particle during 3D refinement; the molecular weight of CV capsid is ∼6 MDa while Fabs are only 50 kDa. pAb-centered subparticles were subjected to a round of 3D refinement with mask around the immune complex and restricted angular search space (local angular searches only). Following refinement, each subset of particles was then subjected to one round of 3D classification with a spherical mask (80 Å diameter) centered on each respective pAb (—skip_align, T 16, K 5). The highest quality classes based on structural features and estimated resolution were selected for the next round of classification. Here, 3D classification was performed with a mask surrounding the entire subparticle comprising pAb and part of CV capsid (—skip_align, T 16, K 3). The highest quality classes based on structural features and estimated resolution were selected and subjected to 3D refinement (same settings as in the previous refinement step). The maps from 3D refinement were postprocessed in Relion. Postprocessing consisted of B-factor sharpening, MTF correction, and solvent masking. The resulting maps were used for model building and submission to EM Data Bank (accession IDs EMD-26072, EMD-26069, EMD-26068, EMD-26070, EMD-26071). Data processing workflows and relevant information are presented in the Supplementary Material Appendix—Fig. S2. Data processing information is presented in the Supplementary Material Appendix—Table S2.

### Model building and refinement

Atomic models were built into postprocessed maps from Relion. CV-A21 structure from PDB entry 1z7s (23) was used as an initial model for the antigen. Only the protomers proximal to the pAb epitope were built into the reconstructed maps. Initial pAb model (Fv-domain only) was generated from PDB entry 3i9g (25) by mutating all of the amino acid residues to alanine. UCSF Chimera (24) was used to dock the antigens/pAbs into the reconstructed maps and create the initial models. The models were then relaxed by iterating manual refinement steps in Coot (42) and automated refinement steps in Rosetta (43). The number of residues in each pAb model was adjusted to best recapitulate the structural features within each cryoEMPEM map. Structural homology to published mouse antibody structures [primarily 3i9g (25)] was used to assign antibody heavy (H) and light (L) chains and individual CDR loops. The principal factors for discrimination are described previously (11). MolProbity (44) and EMRinger (45) metrics were used to assess model quality. The final statistics are shown in the Supplementary Material Appendix—Table S2. All models were submitted to the Protein Data Bank (accession IDs: 7TQU, 7TQS, 7TQT).

## Supplementary Material

pgac253_Supplemental_File

## Data Availability

All data needed to evaluate the conclusions in the paper are present in the manuscript and/or the Extended Data. 3D maps have been deposited to the Electron Microscopy Data Bank (http://www.emdatabank.org/) with accession numbers EMD-26065, EMD-26072, EMD-26069, EMD-26068, EMD-26070, and EMD-26071. Atomic models have been deposited to the Protein Data Bank (http://www.rcsb.org/) with accession numbers 7TQU, 7TQS, and 7TQT. The manuscript text is also available as preprint on bioRxiv (doi: https://doi.org/10.1101/2022.01.31.478566).
