# Supplementary material for: High-resolution structural analysis of enterovirus-reactive polyclonal antibodies in complex with whole virions
Source: PNAS Nexus. 2022 Nov 4;1(5):pgac253. doi: 10.1093/pnasnexus/pgac253 (PMC9802058; doi:10.1093/pnasnexus/pgac253)
Supplement: pgac253_Supplemental_File [file pgac253_supplemental_file.docx]

**Supplementary Information for:**

**Title:**

**High-resolution structural analysis of enterovirus-reactive polyclonal antibodies in complex with whole virions**

**Authors:**

Aleksandar Antanasijevic^1,2^, Autumn J. Schulze^3^, Vijay S. Reddy^1,#^, Andrew B. Ward^1,2,#^

**Affiliations:**

1. Department of Integrative, Structural and Computational Biology, The Scripps Research Institute, La Jolla, CA 92037, USA.
2. International AIDS Vaccine Initiative Neutralizing Antibody Center, and Scripps Consortium for HIV/AIDS Vaccine Development (CHAVD), The Scripps Research Institute, La Jolla, CA 92037, USA.
3. Department of Molecular Medicine, Mayo Clinic College of Medicine, Rochester, MN 55905, USA.

# - Correspondence should be addressed to Andrew Ward (andrew@scripps.edu) and Vijay S. Reddy (reddyv@scripps.edu)

**This PDF file includes:**

Tables S1 to S3

Figures S1 to S7

**Table S1.** **Cryo-EM data collection information**

|  | **CV-A21**  **+**  **Ag-specific mouse pAb** |
| --- | --- |
| **Microscope** | Titan Krios |
| **Voltage (kV)** | 300 |
| **Detector** | Gatan K2 Summit |
| **Recording mode** | Counting |
| **Magnification** | 130,000 |
| **Movie micrograph pixel size** | 1.045 |
| **Dose rate (e^−^/Å^2^/s)** | 5.55 |
| **No. of frames per movie micrograph** | 45 |
| **Frame exposure time (ms)** | 200 |
| **Movie micrograph exposure time (s)** | 9.0 |
| **Total dose (e^−^/Å^2^)** | 49.95 |
| **Grid Type** | Quantifoil R 2/1 |
| **Under focus range (µm)** | 0.8 – 1.8 |
| **Number of movie micrographs** | 3,862 |

**Table S2. Map and model refinement information**

| **Complex ID** | **pAbC1-1** | **pAbC1-2** | **pAbC2-1** | **pAbC2-2** | **pAbC2-3** |
| --- | --- | --- | --- | --- | --- |
| **Antigen** | **CV-A21** | | | | |
| **Number of picked particles** | 22,937 | | | | |
| **Particles after 2D classification** | 22,245 | | | | |
| **Particles after symmetry expansion** | 1,334,700 | | | | |
| **Particles in the final map** | 19,748 | 7,792 | 29,282 | 22,044 | 12,654 |
| **Map symmetry** | C1 | C1 | C1 | C1 | C1 |
| **Map sharpening B-factor** | -65.0 | -100.0 | -55.0 | -40.0 | -45.0 |
| **Map Resolution** | 3.8 | 12.0 | 3.9 | 4.6 | 4.1 |
| **EMDB ID** | 26072 | 26069 | 26068 | 26070 | 26071 |
| **Residues** |  |  |  |  |  |
| **Amino acids** | 2758 | N/A | 4451 | N/A | 4418 |
| **Ligands (MYR)** | 3 | N/A | 5 | N/A | 5 |
| **RMSD Bonds (4𝛔)** | 0.020 | N/A | 0.021 | N/A | 0.020 |
| **RMSD Angles (4𝛔)** | 1.740 | N/A | 1.743 | N/A | 1.766 |
| **Ramachandran** |  |  |  |  |  |
| **Outliers (%)** | 0.00 | N/A | 0.00 | N/A | 0.00 |
| **Allowed (%)** | 2.20 | N/A | 1.84 | N/A | 2.22 |
| **Favored (%)** | 97.80 | N/A | 98.16 | N/A | 97.78 |
| **Rotamer outliers (%)** | 0.00 | N/A | 0.00 | N/A | 0.00 |
| **Clash score** | 0.85 | N/A | 1.07 | N/A | 1.60 |
| **Molprobity score** | 0.81 | N/A | 0.81 | N/A | 0.96 |
| **EMRinger score** | 3.42 | N/A | 3.31 | N/A | 2.35 |
| **PDB ID** | 7TQU | N/A | 7TQS | N/A | 7TQT |

**Table S3.** Sequence alignment of capsid-forming polyprotein across enterovirus C CV-A viral strains

CV-A21 MGAQVSTQKTGAHENQNVAANGSTINYTTINYYKDSASNSATRQDLSQDPSKFTEPVKDL 60

CV-A11_(AAQ02676.1) MGAQVSSQKVGAHENTNVATGGSTVNYTTINYYKDSASNAASKQDFSQDPSKFTEPVKDI 60

CV-A13_(AAQ02677.1) MGAQVSSQKVGAHENTNVATGGSTVNYTTINYYKDSASNAASKQDFSQDPSKFTEPVKDV 60

CV-A17_(AAQ02679.1) MGAQVSSQKVGAHENTNVATGGSTVNYTTINYYKDSASNAASKQDFSQDPSKFTEPVKDI 60

CV-A18_(AAQ02680.1) MGAQVSSQKVGAHENTNVATGGSTVNYTTINYYKDSASNAASKQDFSQDPSKFTEPVKDV 60

CV-A20_(AAQ02682.1) MGAQVSSQKVGAHENTNVATGGSTVNYTTINYYKDSASNAASKQDFSQDPSKFTEPVKDI 60

CV-A24_(ABM54551.1) MGAQVSSQKVGAHENTNVATGGSTVNYTTINYYKDSASNAASKQDFSQDPSKFTEPVKDI 60

CV-A1_(AAQ02675.1) MGAQVSTQKSGSHENQNVAAGGSTINYTTINYYKDSASNSASKQDFSQDPSKFTEPVKDI 60

CV-A19_(AAQ02681.1) MGAQVSTQKSGSHENQNIAAGGSTINYTTINYYKDSASNSAAKQDFSQDPSKFTEPVKDI 60

CV-A22_(AAQ02683.1) MGAQVSTQKSGSHENQNIAASGSTINYTTINYYKDSASNSAAKQDFSQDPSKFTEPVKDI 60

|-----------------------------VP4---------------------------

CV-A21 MLKTAPALNSPNVEACGYSDRVRQITLGNSTITTQEAANAIVAYGEWPTYINDSEANPVD 120

CV-A11_(AAQ02676.1) MLKSAPALNSPNIEACGYSDRVMQLTLGNSTITTQEAANSVVAYGEWPSYLSDKEANPVD 120

CV-A13_(AAQ02677.1) LIKSAPALNSPNIEACGYSDRVMQLTLGNSTITTQEAANSVVAYGVWPSYLSDKDANPVD 120

CV-A17_(AAQ02679.1) MLKSAPALNSPNIEACGYSDRVMQLTLGNSTITTQEAANSVVAYGRWPSYLSDREANPVD 120

CV-A18_(AAQ02680.1) LIKSAPALNSPNIEACGYSDRVMQLTLGNSTITTQEAANSVVAYGVWPSYLSDKDANPVD 120

CV-A20_(AAQ02682.1) MLKSAPALNSPNVEACGYSDRVLQLTLGNSTITTQEAANSVVGYGQWPTYLNAKDANPVD 120

CV-A24_(ABM54551.1) MLKSAPALNSPNVEACGYSDRVRQITLGNSTITTQEAANAVVAYGEWPSYLDDREANPVD 120

CV-A1_(AAQ02675.1) MIKTAPALNSPNIEACGYSDRVLQLTLGNSTITTQEAANSVIAYGEWPSFLSDKEANPVD 120

CV-A19_(AAQ02681.1) IVKTAPALNSPNIEACGYSDRVLQLTLGNSTITTQEAANAVVAYGEWPRFINDQEANPVD 120

CV-A22_(AAQ02683.1) MVKTAPALNSPNIEACGYSDRVLQLTLGNSTITTQEAANAVVAYGEWPSFLSDKEANPVD 120

---VP4--|-----------------------VP2-------------------------

CV-A21 APTEPDVSSNRFYTLESVSWKTTSRGWWWKLPDCLKDMGMFGQNMYYHYLGRSGYTIHVQ 180

CV-A11_(AAQ02676.1) QPTEPEVSACRFYTLDTVTWSKSSKGWWWKLPDALKDMGLFGQNMYYHYLGRSGYTVHVQ 180

CV-A13_(AAQ02677.1) QPTEPDVSACRFYTLDTVEWDKESKGWWWKLPDALKDMGLFGQNMYYHYLGRSGYTVHVQ 180

CV-A17_(AAQ02679.1) QPTEPDVAACRFYTLESVMWSKESRGWWWKLPDALKDMGLFGQNMFYHYLGRSGYTIHVQ 180

CV-A18_(AAQ02680.1) QPTEPDVSACRFYTLETVEWDRESKGWWWKLPDALKDMGLFGQNMYYHYLGRSGYTVHVQ 180

CV-A20_(AAQ02682.1) QPTEPDVSACRFYTLQSVEWKTESKGWWWKLPDALKDMGLFGQNMYYHYLGRSGYTVHVQ 180

CV-A24_(ABM54551.1) APTEPDVSSNRFYTLDSVQWKSTSRGWWWKLPDALKDMGMFGQNMYYHYLGRSGYTVHVQ 180

CV-A1_(AAQ02675.1) APTEPDASSNRFYTLDSKPWAADSRGWWWKLPDALKDMGMFGQNMFYHYLGRAGYTVHVQ 180

CV-A19_(AAQ02681.1) APTEPDASANRFYTLDSVDWGADSKGWWWKLPDALKDMGMFGQNMYYHYLGRAGYTVHVQ 180

CV-A22_(AAQ02683.1) APTEPDASANRFYTLESITWEKSSRGWWWKLPDALKDMGMFGQNMFYHYLGRAGYTVHVQ 180

------------------------------VP2---------------------------

CV-A21 CNASKFHQGALGVFLIPEFVMACNTESKTSYVSYINANPGERGGEFTNTYNPSNTDASEG 240

CV-A11_(AAQ02676.1) CNASKFHQGALGVFAIPEYCMACNTDAKTNYVSYVQANPGEAGGVFTDMYNPS-SETTGA 239

CV-A13_(AAQ02677.1) CNASKFHQGTLGVFAVPEYCLAGDSNSKTNYTSYVNANPGEAGGKFVSTFTPD-TGTSPK 239

CV-A17_(AAQ02679.1) CNASKFHQGTLGVFAIPEYCLAGDSDVKNSYTLYVNANPGERGGTFTDKFTAS-SRTNPT 239

CV-A18_(AAQ02680.1) CNASKFHQGTLGVFAVPEYCLAGDSNSKTNYTSYINANPGERGGTFVSTFTPD-SGAVPK 239

CV-A20_(AAQ02682.1) CNASKFHQGALGVFAVPEYCLAGDSDVKNSYTTYKNANPGETGGVFVDSFTAT-TQS--T 237

CV-A24_(ABM54551.1) CNASKFHQGTLGVFAIPEYVMACNTETKTSYVSYVNANPGERGGVFTSTYNPS-TDAAEG 239

CV-A1_(AAQ02675.1) CNASKFHQGTLLVAAIPEFMMGSNTDTNTGGITYEKANPGEVGGTFQKTATLT-T-GDGK 238

CV-A19_(AAQ02681.1) CNASKFHQGTLFVAAIPEYMMASNSGTNTGGIIYEFANPGEAGGRFSSTFTPD-TQAPGK 239

CV-A22_(AAQ02683.1) CNASKFHQGALIVAAIPEFMMGSNTATSTGGVTYANANPGESGGKFSSQFIPS-SDTEAK 239

------------------------------VP2---------------------------

CV-A21 RKFAALDYLLGSGVLAGNAFVYPHQIINLRTNNSATIVVPYVNSLVIDCMAKHNNWGIVI 300

CV-A11_(AAQ02676.1) RKFAAVDYLLGCGVLAGNAFVFPHQIINLRTNNCATLVLPYVNSMAIDCMAKHNNWGIAI 299

CV-A13_(AAQ02677.1) REFQPVDYLFGCGVMAGNAFVFPHQIINLRTNNCATLVLPYVNSLAIDCMAKHNNWGIVI 299

CV-A17_(AAQ02679.1) RKFCAVDYLLGCGVLAGNAFVFPHQIINLRTNNCATLVLPYVNSLAIDSMTKHNNWGIAI 299

CV-A18_(AAQ02680.1) REFQPVDYLFGCGVMAGNAFVFPHQIINLRTNNCATLVLPYVNSLAIDCMAKHNNWGIVI 299

CV-A20_(AAQ02682.1) RKFCPIDYLFGCGVLTGNAFVFPHQIINLRTNNSATLVLPYVNSLAIDCMAKHNNWGLAI 297

CV-A24_(ABM54551.1) RKFAALDYLLGCGVLAGNAFVFPHQIINLRTNNSATLVLPYVNSLAIDCMAKHNNWGLVI 299

CV-A1_(AAQ02675.1) NSFCPLDWVLGCGVMAGNATVFPHQFINLRTNNSATLVLPYVNSIATDCMAKHNNWGLVV 298

CV-A19_(AAQ02681.1) NKFAPLDWLLGCGVMAGNITVFPHQIINLRTNNCATLVLPYVNSVVTDSMAKHNNWGIVV 299

CV-A22_(AAQ02683.1) NKFAPWDWLLGCGVMAGNITVYPHQIINLRTNNCATLVLPYVNSVATDCMAKHNNWGLVI 299

------------------------------VP2---------------------------

**Table S3.** Sequence alignment of capsid-forming polyprotein across enterovirus C CV-A viral strains (continued)

CV-A21 LPLAPLAFAATSSPQVPITVTIAPMCTEFNGLRNITVPVHQGLPTMNTPGSNQFLTSDDF 360

CV-A11_(AAQ02676.1) LPLAELDFAEASSPEIPITITIAPMCCEFNGLRNLTSPAKQGLPVMNVPGSNQFLSSDNF 359

CV-A13_(AAQ02677.1) LPLSKLDYNPDASTKLPITVTIAPMCCEFNGLRNLTIPATQGLPVMSTPGSNQYLTSDNF 359

CV-A17_(AAQ02679.1) IPLSKLDFAPDASVELPITVTIAPMCCEFNGLRNITIPATQGLPVMNTPGSNQYLTADNF 359

CV-A18_(AAQ02680.1) LPLSKLDYNPDASTKLPITVTIAPMCCEFNGLRNLTIPATQGLPVMNTPGSNQYLTSDNF 359

CV-A20_(AAQ02682.1) IPLSKLQFPDTSSTEIPITVTIAPMCCEFNGLRNITVPSTQGLPVMNTPGSNQYLTSDNF 357

CV-A24_(ABM54551.1) LPLCKLDYAPNSSTEIPITVTIAPMCTEFNGLRNITVPATQGLPTMLTPGSSQFLTSDDF 359

CV-A1_(AAQ02675.1) MPVVPLQYSNGASTLVPITITIAPMCCEFNGLRSLTTPYTQGLPVMNTPGSNQFLTTDNF 358

CV-A19_(AAQ02681.1) IPFVKLAYQNGATTKVPITVTIAPMCCEFNGLRSLTAPVTQGLPTMATPGSNQFLTSDNF 359

CV-A22_(AAQ02683.1) MPFVQLDAVKDATQSVPITVTIAPMCCEFNGLRSLTVPVLQGLPTMSTPGSNQFLTSDDF 359

---------------------VP2----------------|---------VP3-------

CV-A21 QSPCALPNFDVTPPIHIPGEVKNMMELAEIDTLIPMNAVDGKVNTMEMYQIPLNDNLSKA 420

CV-A11_(AAQ02676.1) QSPCALPEFDVTPPIHIPGEVRNMMELAEIDTLIPMDLSESKKNTMGMYRVELGSG-KSL 418

CV-A13_(AAQ02677.1) QSPCALPEFDVTQPIFIPGEVKNLMELAEIDTMIPMDLSEGKRNSMDMYRVKISDA-GDR 418

CV-A17_(AAQ02679.1) QSPCALPEFDVTQPIDIPGEVKNLMEIAEIDTMIPLDLSESKKNTMDMYRVQLQASPSNR 419

CV-A18_(AAQ02680.1) QSPCALPEFDVTQPIFIPGEVKNLMELAEIDTMIPMDLSEGKKNTMEMYRVKLSDT-GNR 418

CV-A20_(AAQ02682.1) QSPCALPEFDVTQAINIPGEVKNIMEIAEIDTMIPLNLSDSRKNSMDMYRVPVTTS-ADL 416

CV-A24_(ABM54551.1) QSPCALPNFDVTPPIHIPGEVTNMMELAEIDSMIPMNSVTGKANTMEMYPIPLNDK-GSA 418

CV-A1_(AAQ02675.1) QSPCALPDFDVTPEIHIPGEVKNMMELAEIDSLVPMNAVAGKVNSMEAYQIPIQANQQDN 418

CV-A19_(AAQ02681.1) QSPCALPDFDVTPEIHIPGEVKNMMELAEIDTLIPMNAIAKKVDTMEAYPIPLQAGVQNN 419

CV-A22_(AAQ02683.1) QSPCALPNFDVTPAIHIPGEVKNMMELAEIDTLIPMNAISTNVNKMEAYPIPLQASKPNA 419

------------------------------VP3---------------------------

CV-A21 --PIFCLSLSPASDKRLSHTMLGEILNYYTHWTGSIRFTFLFCGSMMATGKLLLSYSPPG 478

CV-A11_(AAQ02676.1) SKPILCLSLSPASEQRLGYTMLGEILNYYTHWSGSLKFTFLFCGSMMATGKILISYAPPG 478

CV-A13_(AAQ02677.1) NKPILCLTLSPASDPRLSYTMLGEILNYYTHWAGSLKFSFLFCGSMMATGKLLVAYSPPG 478

CV-A17_(AAQ02679.1) DTPILCLSLSPASDPRLSFTMLGEILNYYTHWAGSLKFSFLFCGSMMATGKILVSYAPPG 479

CV-A18_(AAQ02680.1) DKPILCLSLSPASDPRLSYTMLGEILNYYTHWAGSLKFSFLFCGSMMATGKLLVAYSPPG 478

CV-A20_(AAQ02682.1) DKPILCLSLSPASDERLSYTMLGEILNYYTHWAGSIKYTFLFCGSMMATGKLLIAYAPPG 476

CV-A24_(ABM54551.1) D-PIFSISLSPASDKRLQYTMLGEILNYYTHWTGSLRFTFLFCGSMMATGKILLSYSPPG 477

CV-A1_(AAQ02675.1) NKSIFAISLSPAANPRLSYTMLGEILNYYTHWSGSIKFTFLFCGSAMATGKLLLSYSPPG 478

CV-A19_(AAQ02681.1) NQSIFSISLSPAADQRLSRTMLGEILNYYTHWTGSIKFTFLFCGSMMATGKILLSYSPPG 479

CV-A22_(AAQ02683.1) SESIFSISLSPAADERLAHTMLGEILNYYTHWTGSLKFTFLFCGSMMATGKILISYSPPG 479

------------------------------VP3---------------------------

CV-A21 AKPPTNRKDAMLGTHIIWDLGLQSSCSMVAPWISNTVYRRCARDDFTEGGFITCFYQTRI 538

CV-A11_(AAQ02676.1) AKPPTTRREAMLGTHVIWDIGLQSSATMVVPWISNVMYRRCVKDDFTEGGYISMFYQTKI 538

CV-A13_(AAQ02677.1) AQPPQDRKAAMLGTHVIWDIGLQSSCTMVVPWISNTSYRRTVKDDFTEGGYISMFYQTRV 538

CV-A17_(AAQ02679.1) AQPPKTRKDAMLGTHLIWDIGLQSSCTMVVPWISNTAYRRTIKDDFTEGGYISMFYQTKI 539

CV-A18_(AAQ02680.1) AQPPQDRKAAMLGTHVIWDIGLQSSCTMVVPWISNTSYRRTAKDDFTEGGYISMFYQTRI 538

CV-A20_(AAQ02682.1) AKPPRTRKEAMLGTHVIWDVGLQSSCTMVVPWISNTAYRRTVEDDFTEGGYISMFYQTKI 536

CV-A24_(ABM54551.1) ASPPKTRKDAMLGTHIIWDLGLQSSCTMLAPWISNTVYRRCVKDDFTEAGYITCFYQTRI 537

CV-A1_(AAQ02675.1) AKPPTTRKEAMLGTHIIWDVGLQSSATMVAPWISNVNYRRCVKDDFTEGGYICCFYQTAI 538

CV-A19_(AAQ02681.1) AKPPTTRKEAMLGTHLIWDIGLQSSATMVAPWISNVNYRRCVKDDFTEGGYICCFYQTAI 539

CV-A22_(AAQ02683.1) AKPPTTRKEAMLGTHVIWDLGLQSSVTLVAPWISNVNYRRCVRDDFTEGGYICAFYQTAI 539

------------------------------VP3---------------------------

CV-A21 VVPASTPTSMFMLGFVSACPDFSVRLLRDTPHISQSKLIGRTQG-IEDLIDTAIKNALRV 597

CV-A11_(AAQ02676.1) VVPLSTPTTMTLLSFVSACNDFTVRLLRDTTHISQTTK-INTQGPIEEIISTVASNALAL 597

CV-A13_(AAQ02677.1) VVPASTPTSMDILCFISACNDFTVRLLRDTTHITQSAL---PQG-LEDLIQQVASNALQL 594

CV-A17_(AAQ02679.1) VVPASTPTTMDIIGFVSACNDFSVRLLRDTTHIAQTAM---PQG-IEDLIQQVASNALQI 595

CV-A18_(AAQ02680.1) VVPASTPTSMDILCFVSACNDFTVRLLRDTTHISQSAM---PQG-LEDLIQQVATNALSL 594

CV-A20_(AAQ02682.1) VVPASTPTNMDILGFVSACNDFSVRLLRDTTHISQTAM---PQG-IEDLITEVASNALKL 592

CV-A24_(ABM54551.1) VVPSGTPTSMFMLAFVSACPDFSVRLLQDTSHISQTALVARTQG-IEDTIDTVINNALQL 596

CV-A1_(AAQ02675.1) VVPSGTPVTMSMLCFVSACNDFTARLLKDSPHVIQNNAV--AQG-LGDSIEAAIDSITQN 595

CV-A19_(AAQ02681.1) VVPPGAPTDMSMLAFVSACNDFSARLLKDTPFITQQALTVTTQG-IDDIIDNVVTNALKV 598

CV-A22_(AAQ02683.1) IVPPSTPTAMYMLAFVSACNDFSVRLLKDTPFVRQDQY-VNTQG-IEDTIEKVVGDALRV 597

---------------------VP3------------------|-------VP1-------

**Table S3.** Sequence alignment of capsid-forming polyprotein across enterovirus C CV-A viral strains (continued)

CV-A21 SQP---------PSTQSTEATSGVNSQEVPALTAVETGASGQAIPSDVVETRHVVNYKTR 648

CV-A11_(AAQ02676.1) SQP-KPV----DNSVQNTQQSAPVHSQEVPALTAVETGATSDVVPSDLIQTRHVLNVKSR 652

CV-A13_(AAQ02677.1) SQPTRPALPPAEQSVPNTNQTTPEHSKEVPALTAVETGATNPLEPGDTVQTRHVIQTRSR 654

CV-A17_(AAQ02679.1) SQPTRPALPSTE-SLPNTQQSAPSHSQEVPALTAVETGATNPLEPSDTVQTRHVIQTRSR 654

CV-A18_(AAQ02680.1) SQPTRPALPPAEQSVPNTSQTTPEHSKEVPALTAVETGATNPLEPGDTVQTRHVVQTRSR 654

CV-A20_(AAQ02682.1) SQP-KPS---TQQSLPNTSSSEPTHSQEAPALTAVETGATSSVVPADLVQTRHVIQTRSR 648

CV-A24_(ABM54551.1) SQP----QPNKQLTAQSTPSTSGVNSQEVPALTAVETGASGQAVPSDVIETRHVVNYKTR 652

CV-A1_(AAQ02675.1) ALT----------TVQNTTQSGPTHSKEVPALTAVETGATSQVEPGDLIETRHVINMRQR 645

CV-A19_(AAQ02681.1) SMP----------QVQDTQSSGPVNSKEVPALTAVETGATSQVDPSDLIETRHVINNRLR 648

CV-A22_(AAQ02683.1) SMP----------QVANTQPSGPVNSKEVPALTAVETGATSQVTPEDLIETRHVINNRLR 647

------------------------------VP1---------------------------

CV-A21 SESCLESFFGRAACVTILSLTNSSKS---GEEKKHFNIWNITYTDTVQLRRKLEFFTYSR 705

CV-A11_(AAQ02676.1) SESTIESFFARAACVTIMQVDNFNAT-SVEDKRKLFAKWAITYTDTVQLRRKLEFFTYSR 711

CV-A13_(AAQ02677.1) SESTVESFFARGACVTIMGVDNYNETLKGDQKSTLFTTWNITYTDTVQLRRKLEMFTYSR 714

CV-A17_(AAQ02679.1) SESTIESFFARGACVTIMTVENFNAT-EAADKKKLFATWNITYTDTVQLRRKLEMFTYSR 713

CV-A18_(AAQ02680.1) SESTVESFFARGACVTIMGVDNYNESLTSSQKSTLFATWNITYTDTVQLRRKLEMFTYSR 714

CV-A20_(AAQ02682.1) SESTVESFFARGACVTIMSVENYNET--AIAESKLFTKWNITYTDTVQLRRKLEMFTYSR 706

CV-A24_(ABM54551.1) SESTLESFFGRSACVTIIEVENFNAT-SEADKRKQFTTWPITYTNTVQLRRKLEFFTYSR 711

CV-A1_(AAQ02675.1) SEASIESFFGRSACVAILGLSNAKPT--DTNTKQLFKTWRISYLETHQLRRKLEFFTYSR 703

CV-A19_(AAQ02681.1) SECTIESFFGRSACVAIIGLSNQKPT--SDNAAKLFATWKISYLDMYQLRRKLEFFTYSR 706

CV-A22_(AAQ02683.1) SECTVEAFFGRSACVAILGVVNKKPD--TTNAKDLFTTWRITYLQTYQLRRKLELFTYSR 705

------------------------------VP1---------------------------

CV-A21 FDLEMTFVFTENYPSTASGEVRNQVYQIMYIPPGAPRPSSWDDYTWQSSSNPSIFYMYGN 765

CV-A11_(AAQ02676.1) FDLEMTFVLTERYYSQSSGHARSQVYQIMYVPPGAPTPSAWDDYTWQTSSNPSIFFTTGN 771

CV-A13_(AAQ02677.1) FDIEFTFVVTERYYSSNSGHALNQVYQIMYVPPGAPVPKKWDDYTWQTSSNPSIFYTYGS 774

CV-A17_(AAQ02679.1) FDIEFTFVTTERYYASNSGHARNQVYQLMYVPPGAPVPQQWDDYTWQTSSNPSVFYTYGD 773

CV-A18_(AAQ02680.1) FDIEFTFVVTERYYSSNSGHALNQVYQIMYVPPGAPIPKKWDDYTWQTSSNPSIFYTYGT 774

CV-A20_(AAQ02682.1) FDIEFTFVVTERYHSANSGHALNQVYQIMYVPPGAPVPQRWDDYTWQTSSNPSVFYTYGT 766

CV-A24_(ABM54551.1) FDLEMTFVVTERYYASNTGHARNQVYQIMYIPPGAPQPTAWDDYTWQSSSNPSVFYTYGS 771

CV-A1_(AAQ02675.1) FDLEMTIVITERVFNAVNVPLRNYVYQIMYVPPGAPEPQSWDDYTWQSSTNPSIFYTTGN 763

CV-A19_(AAQ02681.1) FDLELTFVISERFFTSTSAAARDYVYQIMYIPPGAPIPQVWDDYTWQSSTNPSIFYTTGN 766

CV-A22_(AAQ02683.1) FDLELTFVITERYFSGTAATTRDYVYQIMYVPPGAPIPNTWDDYTWQSSTNPSVFYTTGN 765

------------------------------VP1---------------------------

CV-A21 APPRMSIPYVGIANAYSHFYDGFARVPLEGENTDAGDTF-YGLVSINDFGVLAVRAVNRS 824

CV-A11_(AAQ02676.1) APPRISIPFVGIANAYSHFYDGFSRVPLEGETTDTGDAY-YGLTSINDFGTLAVRVVNDY 830

CV-A13_(AAQ02677.1) APPRISIPFVGIANAYSHFYDGYATVPLKTDTTDSGAAY-YGAVSINDFGLLAVRVVNEH 833

CV-A17_(AAQ02679.1) APARISIPFVGIANAYSHFYDGYAVVPLKDSTQDAGAAY-YGATSINDFGMLAVRVVNEF 832

CV-A18_(AAQ02680.1) APPRISIPFVGITNAYSHFYDGYATVPLKTDTTDPGAAF-YGAVSINDFGLLAVRVVNEH 833

CV-A20_(AAQ02682.1) APARISIPYVGIANAYSHFYDGFAKVPIEGETSDPGDAY-YGATSINDFGILAIRVVNEH 825

CV-A24_(ABM54551.1) APPRMSIPYVGIANAYSLFYDGFARVPLKDETADSGDTF-YGLVTINDFGILAIRVVNEF 830

CV-A1_(AAQ02675.1) APPRVSIPFVGIGSAYSHFYDGFSQIPL--DSISAGASNKYGYTSINDFGTLAIRIVNEY 821

CV-A19_(AAQ02681.1) ACPRVSIPFVGIGAAYSHFYDGFSLVPF--NTIDAGASNRYGYTTINDFGTMAIRIVNEY 824

CV-A22_(AAQ02683.1) ASPRMSIPFVGIGAAYAHFYDGFSVVPF--NQIDAGASNKYGYSSIKDFGTLAVRIVNEF 823

------------------------------VP1---------------------------

CV-A21 NPHTIHTSVRVYMKPKHIRCWCPRPPRAVLYRGEGVDMISSAILPLAKVDSITTF

CV-A11_(AAQ02676.1) NPARVETRIRVYMKPKHVRVWCPRPPRAVSYRGPGVDLLSTSVTPLSK-HDLATY

CV-A13_(AAQ02677.1) NPVRVSSKIRVYMKPKHVRVWCPRPPRAVEYYGPGVDYKANTLTPLPI-KNLTTY

CV-A17_(AAQ02679.1) NPARITSKLRVYMKPKHVRVWCPRPPRVVPYFGPGVDYK-DSLTPLST-KALNTY

CV-A18_(AAQ02680.1) NPVRVSSKIRVYMKPKHVRVWCPRPPRAVEYYGPGVDYKANTLTPLPT-KNLTTY

CV-A20_(AAQ02682.1) NPVQVSSKIRVYMKPKHVRVWCPRPPRAVPYFGPGVDYKGDALTPLSR-KDLTTY

CV-A24_(ABM54551.1) NPARITSKIRVYMKPKHVRCWCPRPPRAVPYRGEGVDFNSSSITPLTAVANINTF

CV-A1_(AAQ02675.1) DPVQVDAKARVYIKPKHVRMWCPRPPRAMPYKNSTVDFDPSATV-MTQVADIRTY

CV-A19_(AAQ02681.1) DPVTIDAKVRVYMKPKHIKVWCPRPPRAVAYNGPTVNFNENPHV-MTAVADIRTY

CV-A22_(AAQ02683.1) DPVTIEAKVRVYMKPKHVRVWCPRPPRAVPYQNSSVDFAQNAVA-MNQVATIRTY

---------------------------VP1-------------------------

**
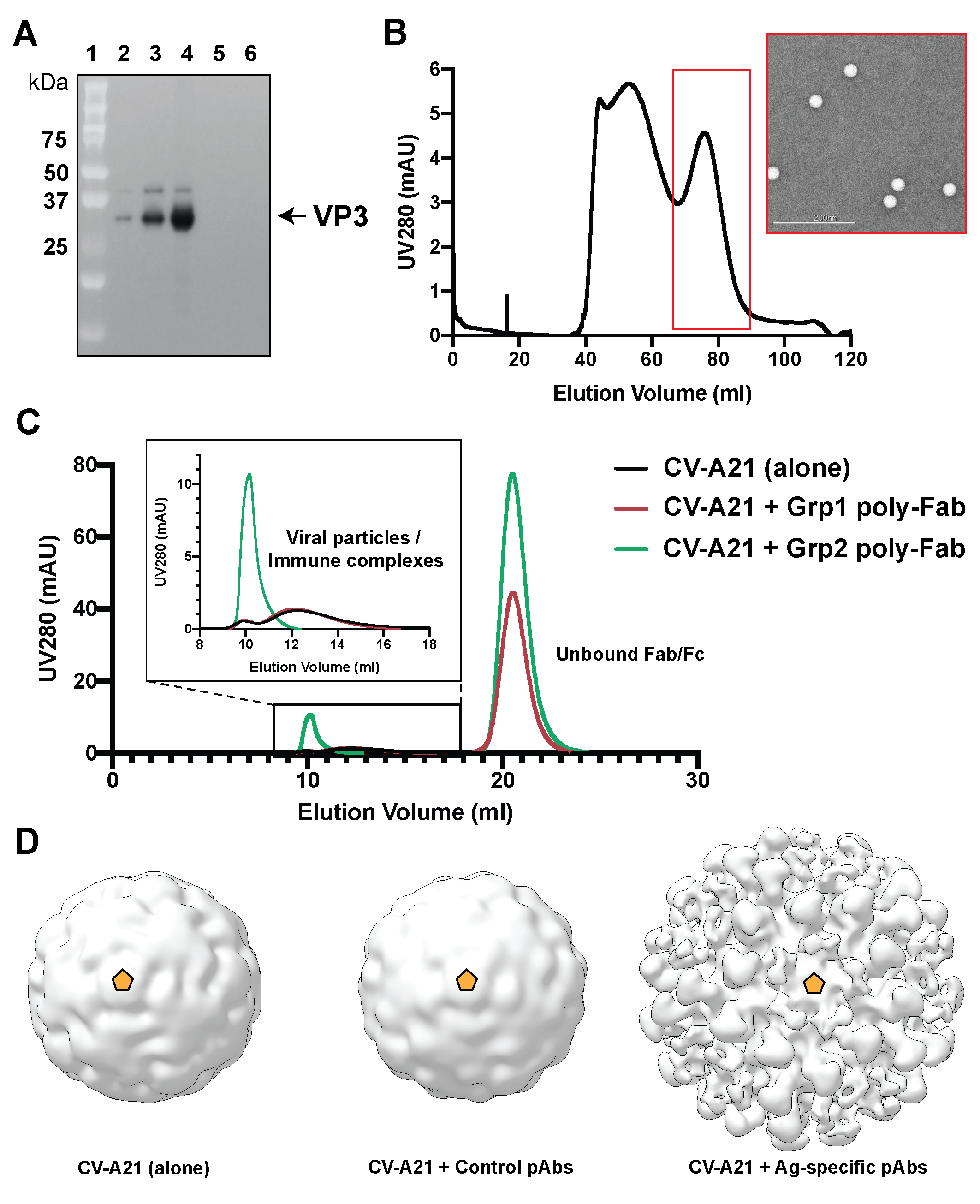
**

**Figure S1. Purification and nsEM characterization of CV-A21 viruses and immune complexes (related to Figure 1). [A]** Western blot obtained using an Enterovirus pan monoclonal antibody L66J (ThermoFisher; MA5-18206); Lane 1: protein ladder; Lane 2: unconcentrated virus; Lane 3: 30% sucrose purified virus; Lane 4: gradient purified virus; Lane 5: PBS gradient purification control; Lane 6: H1-HeLa cell lysate control. **[B]** SEC purification of formaldehyde-treated CV-A21 particles. Sephacryl S-500 HR column was used for the purification step. Elution peak and nsEM micrographs corresponding to CV-A21 are shown in red. **[C]** SEC purification of CV-A21 viral particles complexed with pAb samples from immunized mice (green and red lines). Noncomplexed CV-A21was also ran as a control (black line). Superose 6 increase 10/300 gl column was used for the SEC step. **[D]** 3D maps of CV-A21-containing immune complexes reconstructed with icosahedral symmetry imposed. Forward-most facing 5-fold symmetry axis is indicated using golden-yellow pentamer.


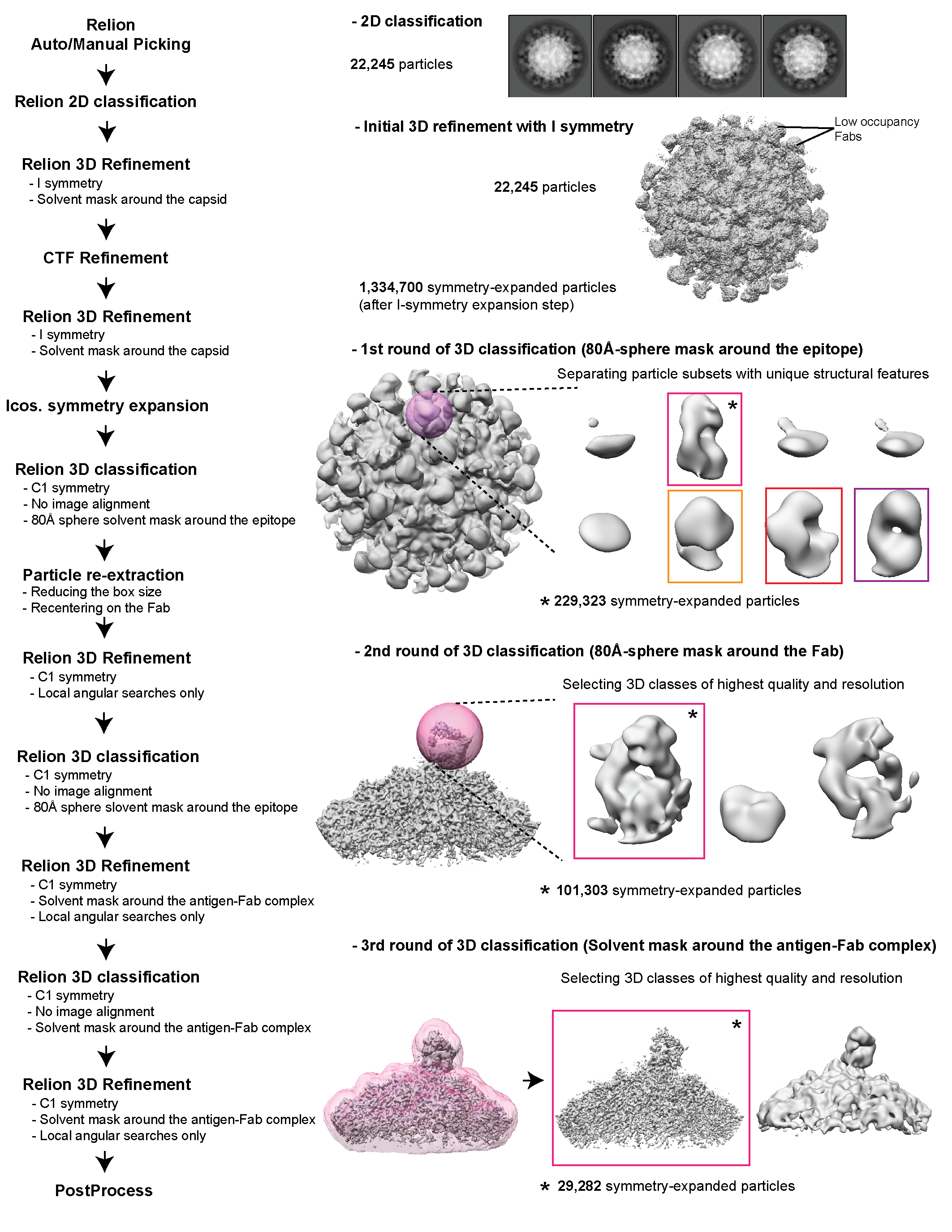


**Figure S2.** **Schematic representation of the focused classification approach used for processing of cryoEMPEM data (related to Figure 2).** Full data processing workflow is shown on the left and the examples of intermediate results are shown on the right.

**
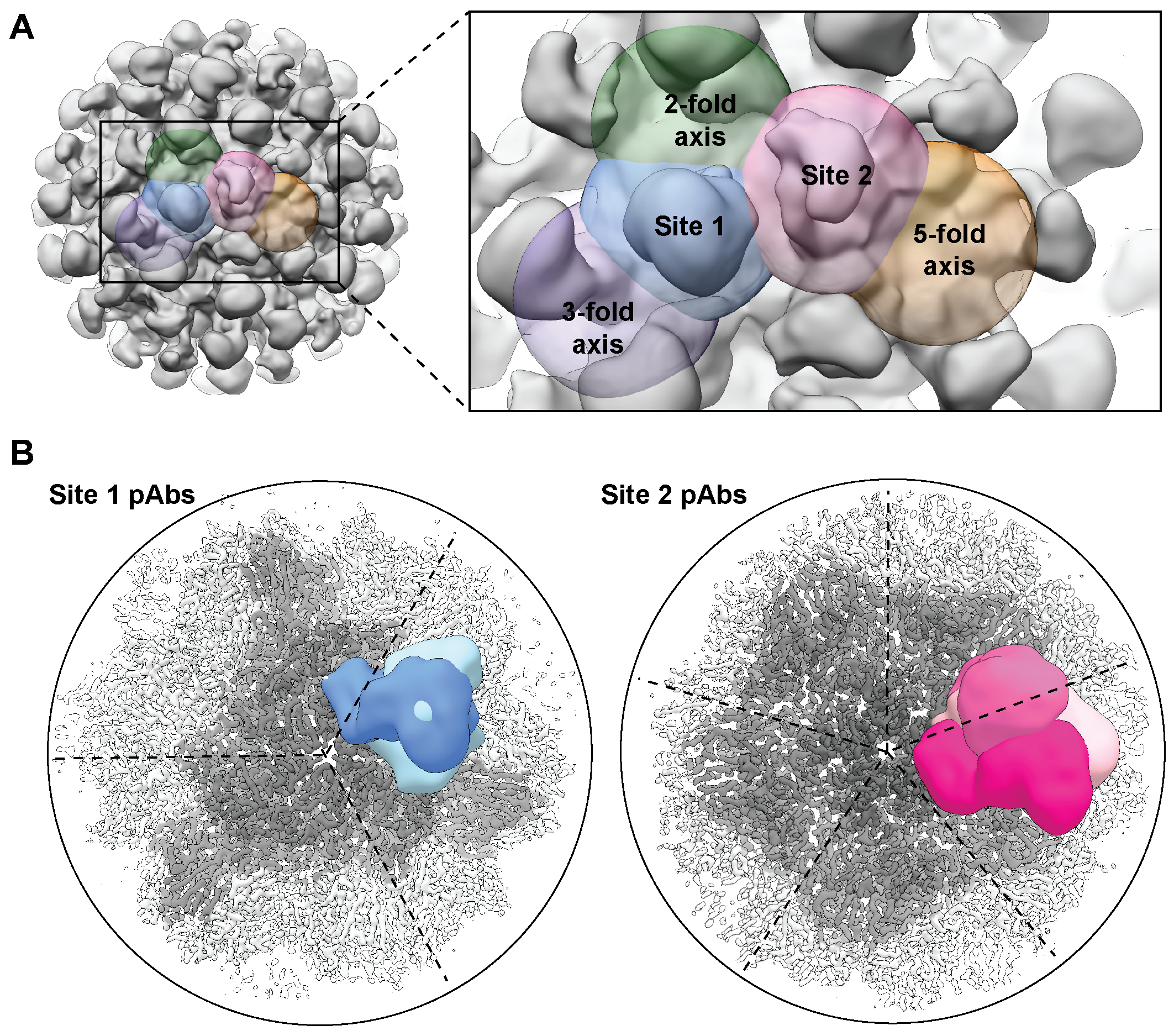
Figure S3. Solvent masks used for data processing and overlay of reconstructed maps (related to Figure 2).** **[A]** The positions of spherical solvent masks used for focused classification of antigen-bound pAbs. **[B]** Overlay of the reconstructed pAbs at Site 1 (left) and Site 2 (right). Different pAbs are shown in different shades of blue (Site 1) and pink (Site 2). pAb-corresponding density in each overlayed map was low-pass filtered to 15Å resolution for clarity. The location of the 3-fold and the 5-fold symmetry axes are indicated in the corresponding panel.


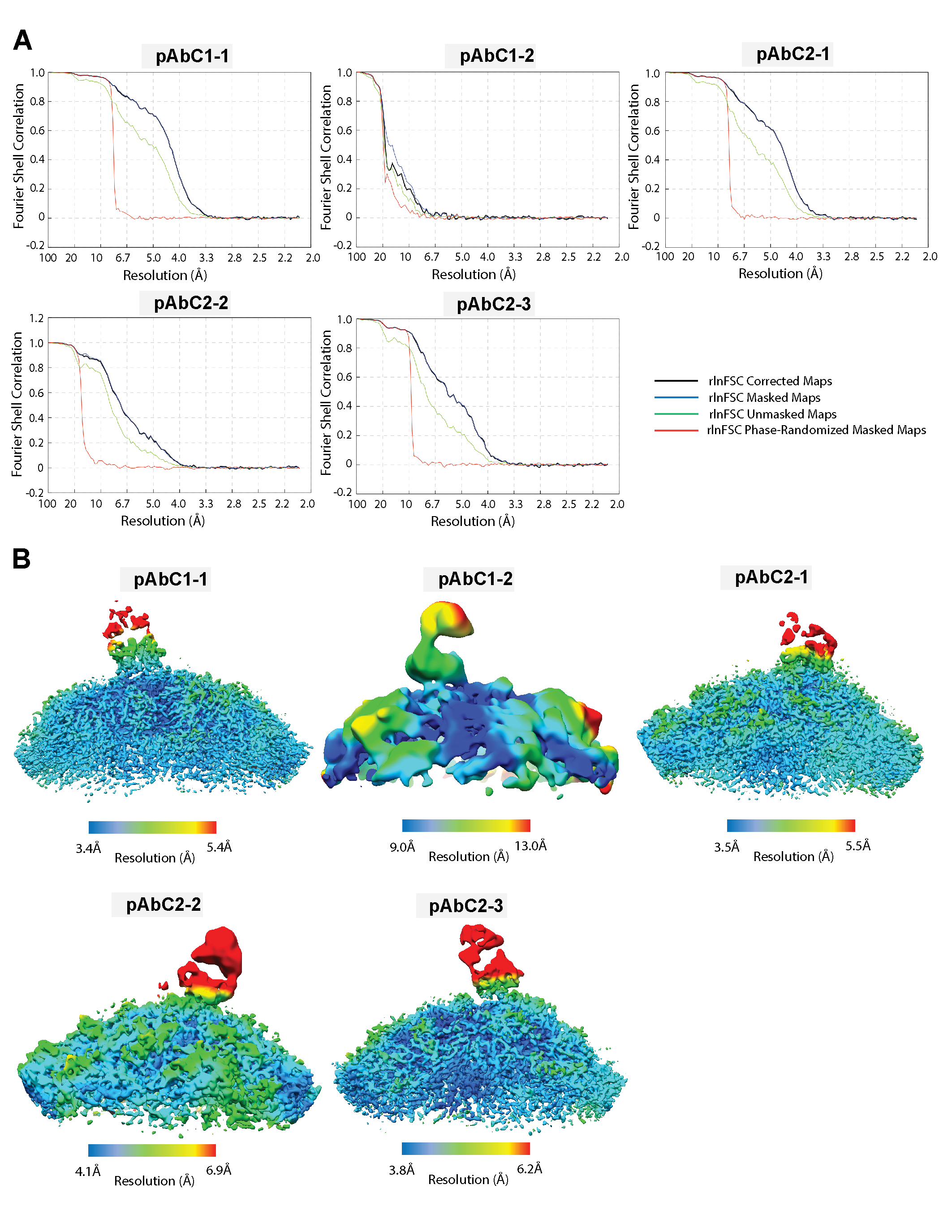


**Figure S4.** **Extended cryoEMPEM data (related to Figure 2).** FSC resolution plots **[A]** and local resolution plots **[B]** for EM maps reconstructed by cryoEMPEM analysis of mouse pAb sample. The plots were generated in Relion/3.0.


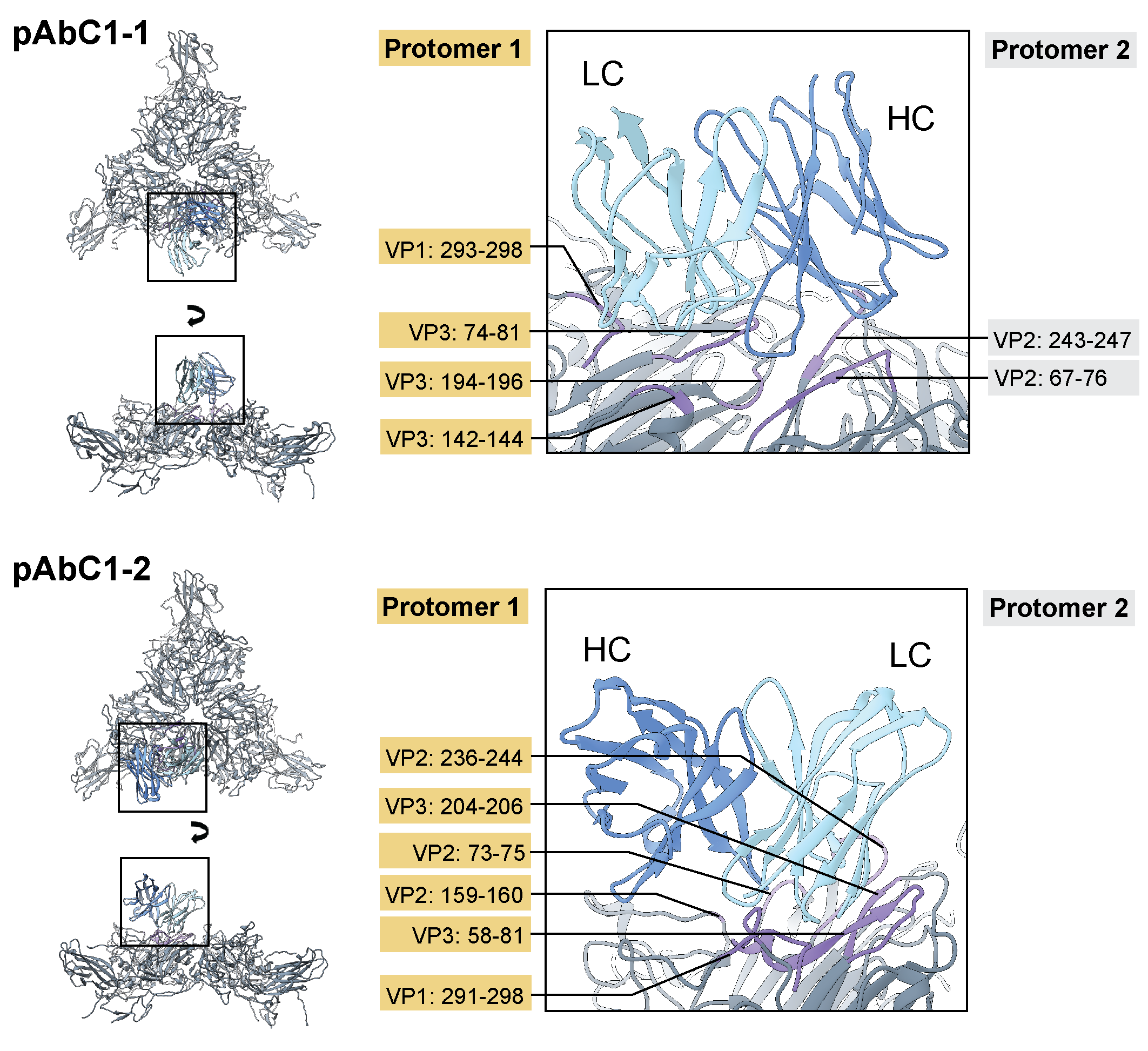


**Figure S5.** **Epitope-paratope interactions formed by Site-1 targeting polyclonal antibodies, pAbC1-1 (top) and pAbC1-2 (bottom) (related to Figure 3).** Ribbon representation used throughout the figure. Full models are presented on the left and close-up views of the epitope-paratope interfaces are shown on the right. Heavy and light chains of each antibody are represented in darker and lighter shades of blue, respectively. Contact residues in each epitope are colored purple, while the rest of the antigen is in dark gray. Residue ranges are indicated on the left and right side of the close-up panel and separated based on the protomer they belong to. For pAbC1-1 we used a refined model with pAb (Fv fragment) represented as poly-Ala pseudo-model. Map resolution was too low to build a model for pAbC1-2 and the presented model was made by docking 3 capsid protomers (each consisting of VP1-4) and a mock mouse Fv fragment (PDB ID: 3i9g) into the pAbC1-2 map.


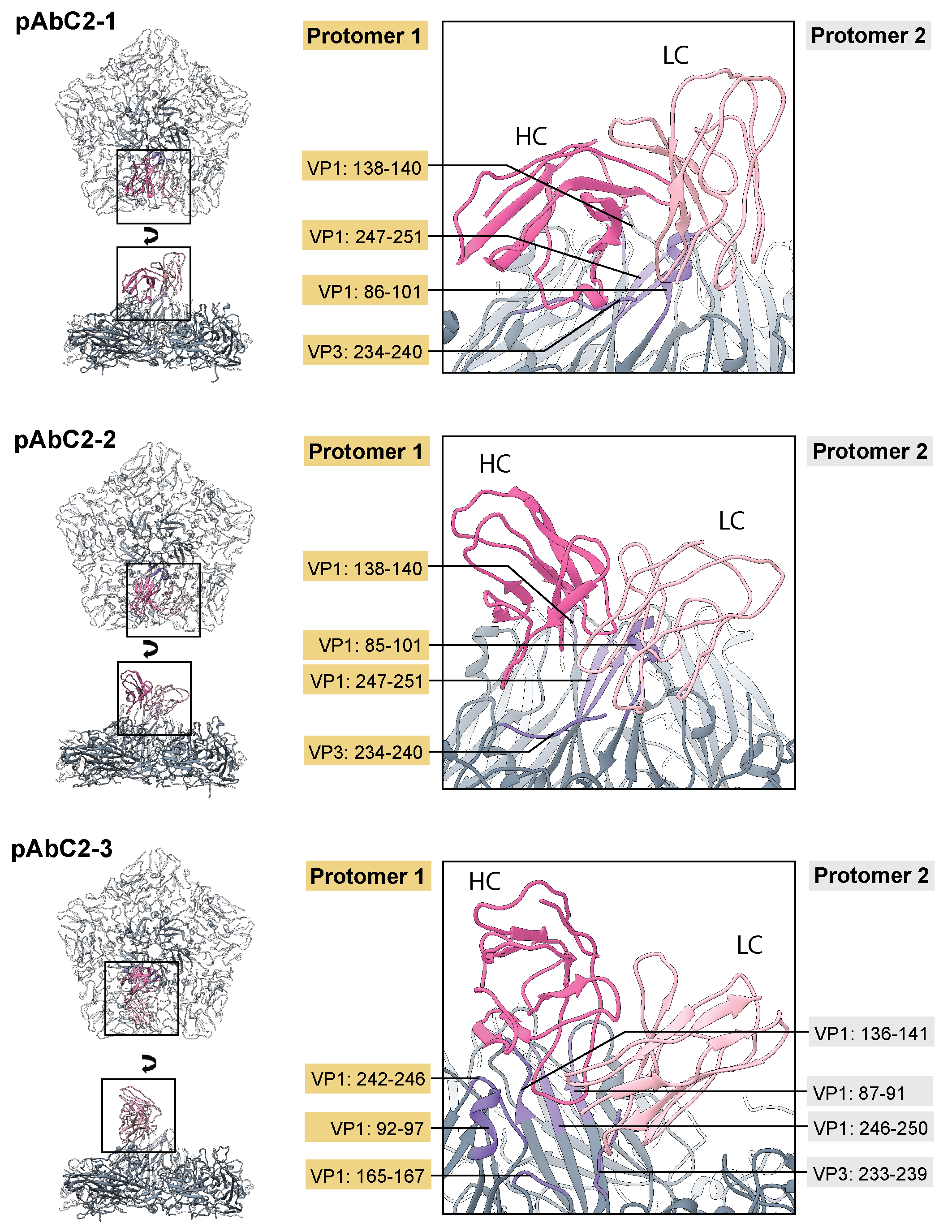


**Figure S6.** **Epitope-paratope interactions formed by Site-1 targeting polyclonal antibodies, pAbC2-1 (top), pAbC2-2 (middle) and pAbC2-3 (bottom) (related to Figure 3).** Ribbon representation used throughout the figure. Full models are presented on the left and close-up views of the epitope-paratope interfaces are shown on the right. Heavy and light chains of each antibody are represented in darker and lighter shades of pink, respectively. Contact residues in each epitope are colored purple, while the rest of the antigen is in dark gray. Residue ranges are indicated on the left and right side of the close-up panel and separated based on the protomer they belong to. For pAbC2-1 and pAbC2-3 we used refined models with polyclonal pAbs (Fv fragment) represented as poly-Ala pseudo-models. Map resolution was too low to build a model for pAbC2-2 and the presented model was made by docking 5 capsid protomers (each consisting of VP1-4) and a mock mouse Fv fragment (PDB ID: 3i9g) into the pAbC2-2 map.


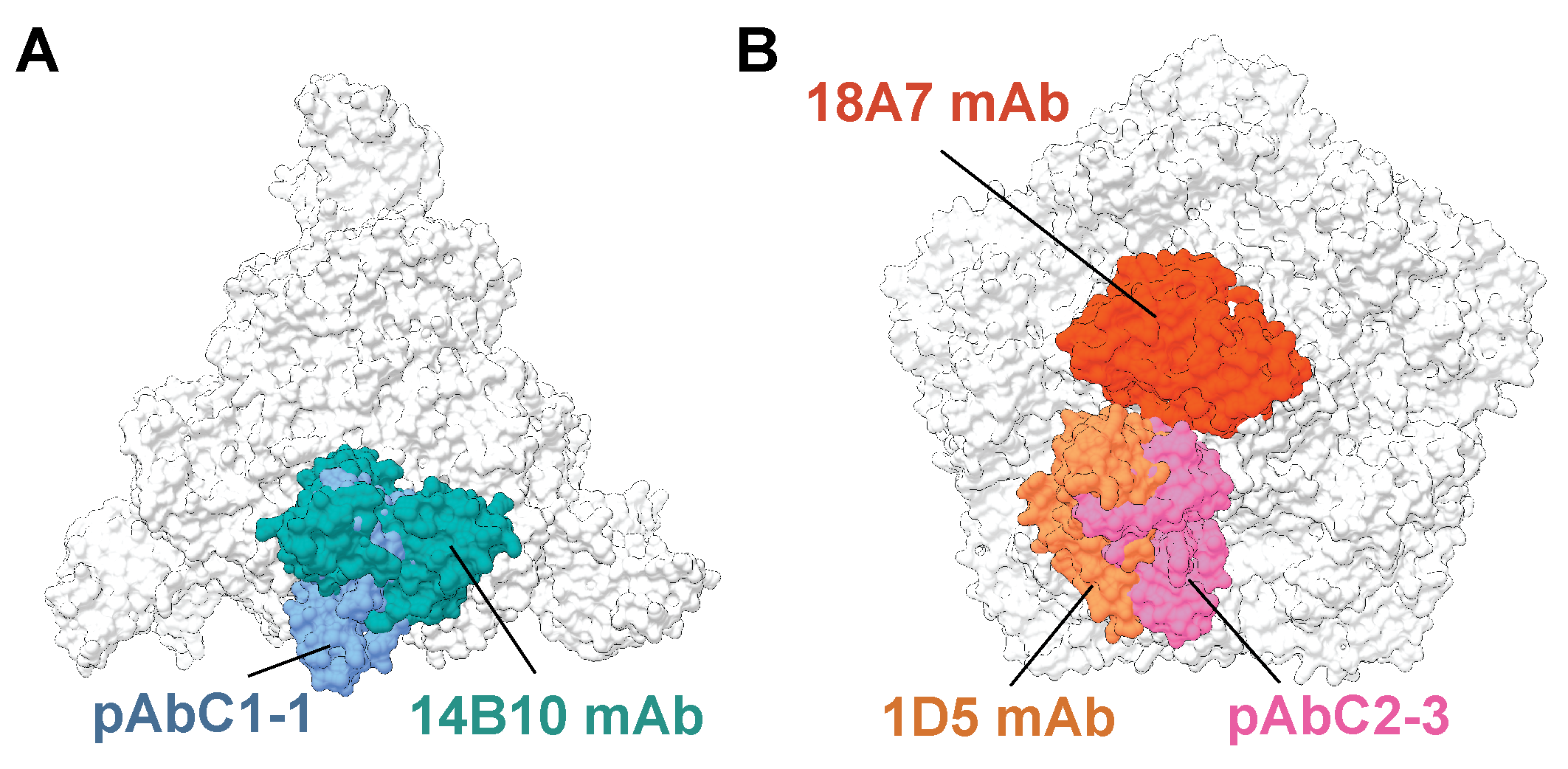


**Figure S7.** **Structural comparison of published CV-specific monoclonal antibodies and polyclonal antibodies recovered in this study (Related to Figure 3). [A]** Overlay of the structures of Site-1 targeting antibodies, pAbC1-1 and 14B10 mAb (ref). **[B]** Overlay of the structures of Site-2 targeting antibodies, pAbC2-3, 1D5 (ref) and 18A7 (ref).
